# Supplementary figures and images for: Probiotics counteract the expression of hepatic profibrotic genes via the attenuation of TGF-β/SMAD signaling and autophagy in hepatic stellate cells
Source: PLoS One. 2022 Jan 20;17(1):e0262767. doi: 10.1371/journal.pone.0262767 (PMC8775563; doi:10.1371/journal.pone.0262767)

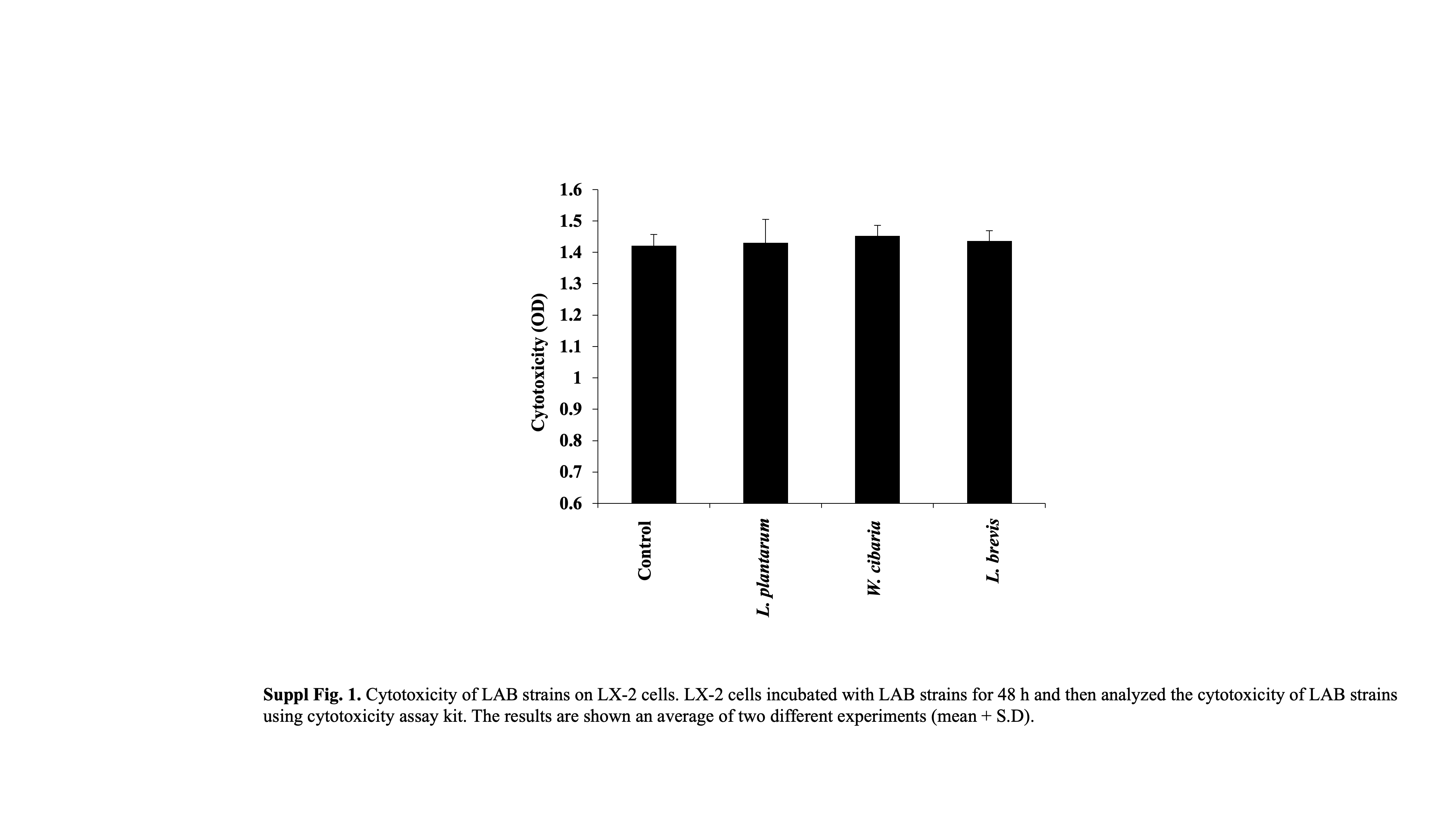

Supplement: S1 Fig — LX-2 cells incubated with LAB strains for 48 h and then analyzed the cytotoxicity of LAB strains using cytotoxicity assay kit. The results are shown an average of two different experiments (mean + S.D). (TIFF) [file pone.0262767.s001.tiff]

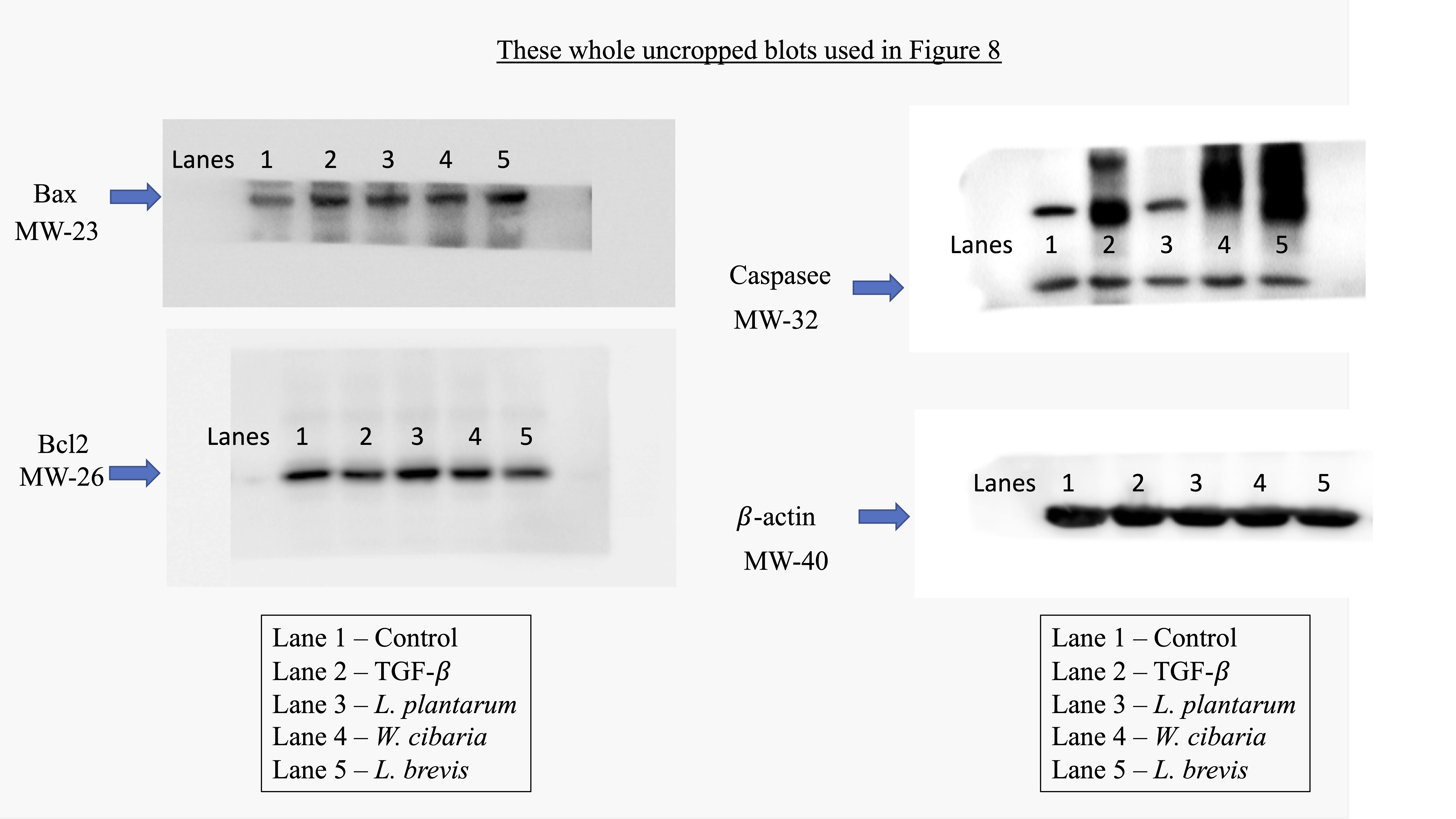

Supplement: S1 Raw images — (ZIP) [file pone.0262767.s003.zip › Uncropped blots_Figure 8.tiff]

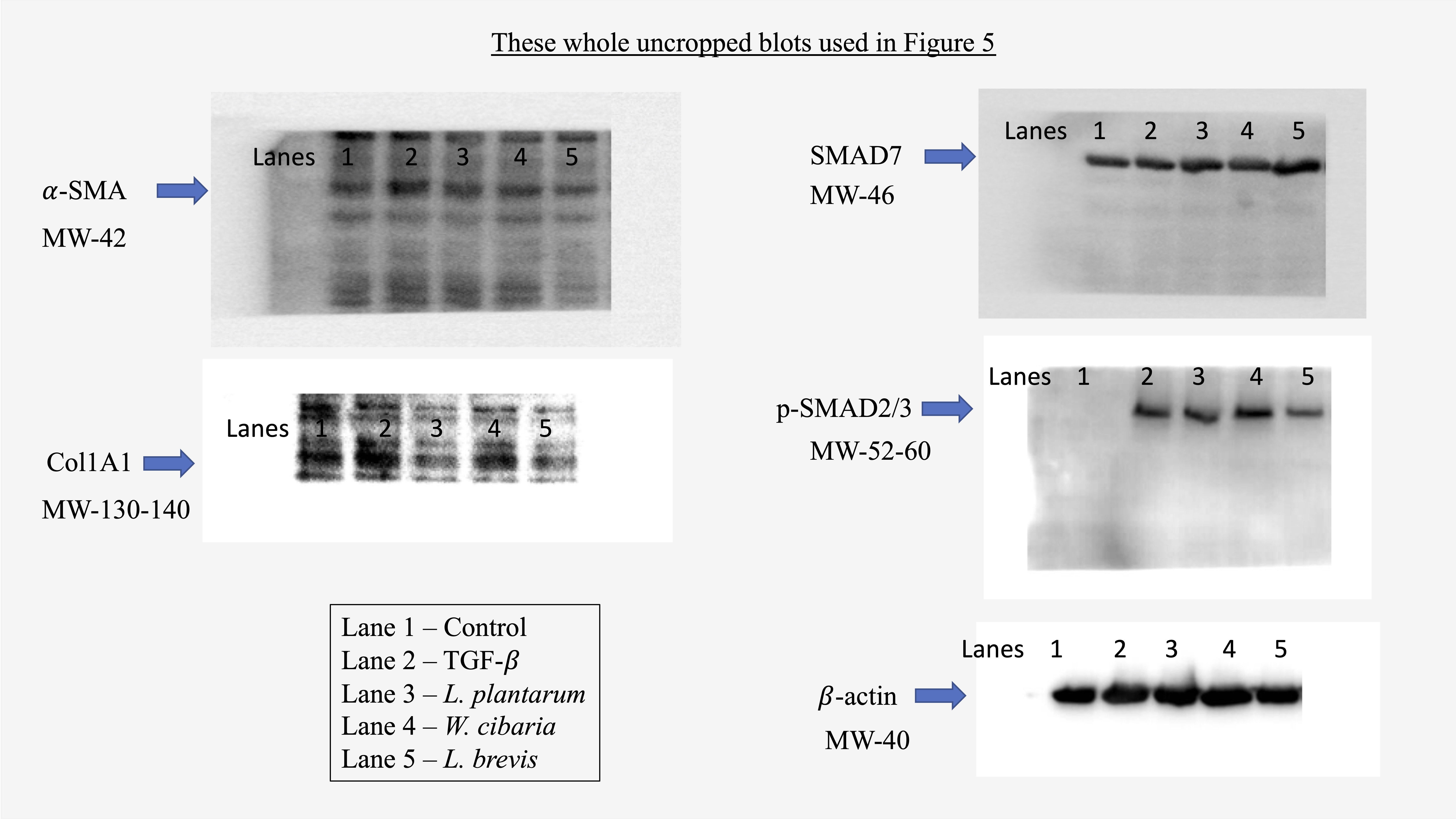

Supplement: S1 Raw images — (ZIP) [file pone.0262767.s003.zip › Uncropped blots_Figure 5.tiff]

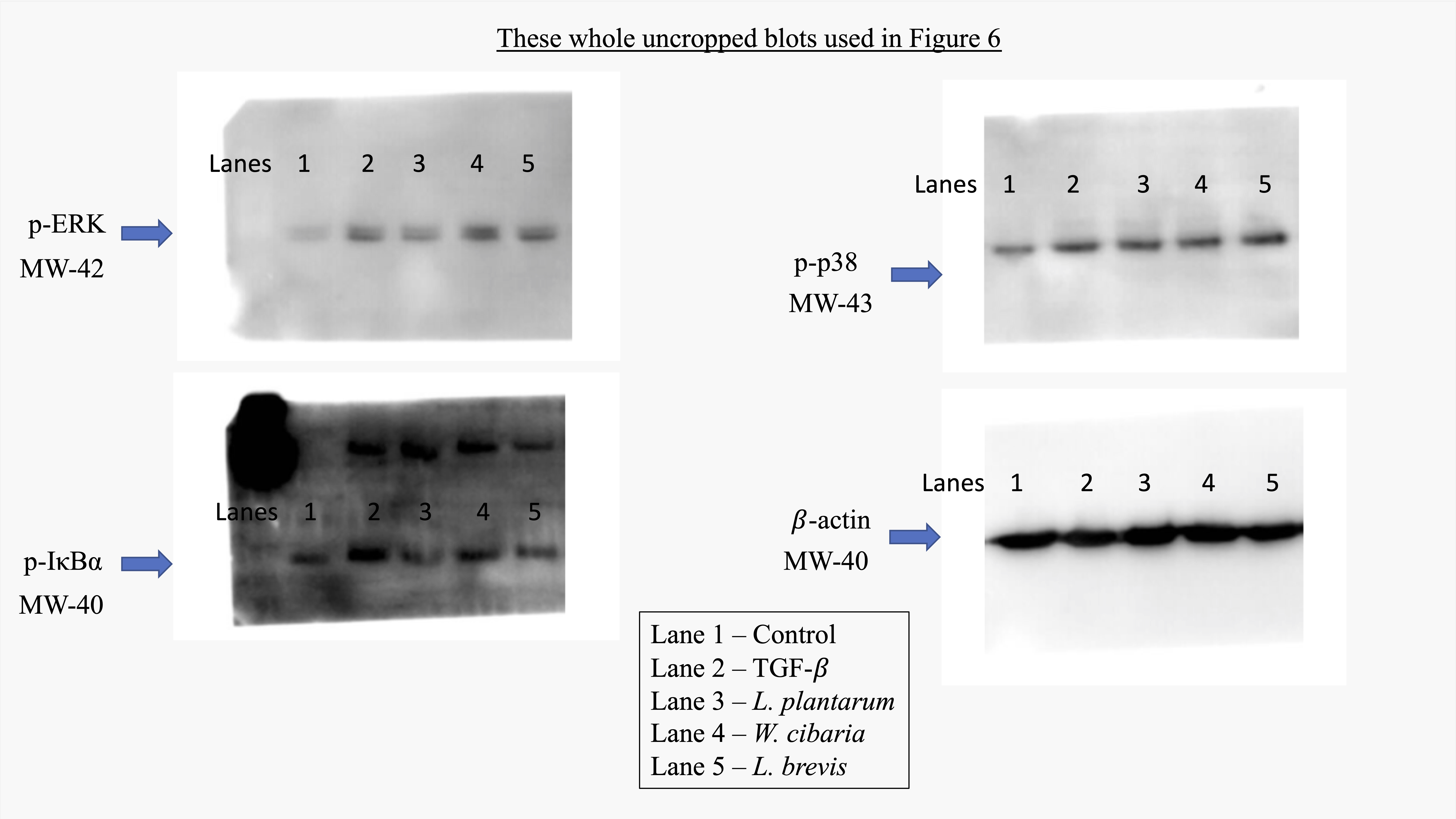

Supplement: S1 Raw images — (ZIP) [file pone.0262767.s003.zip › Uncropped blots_Figure 6.tiff]

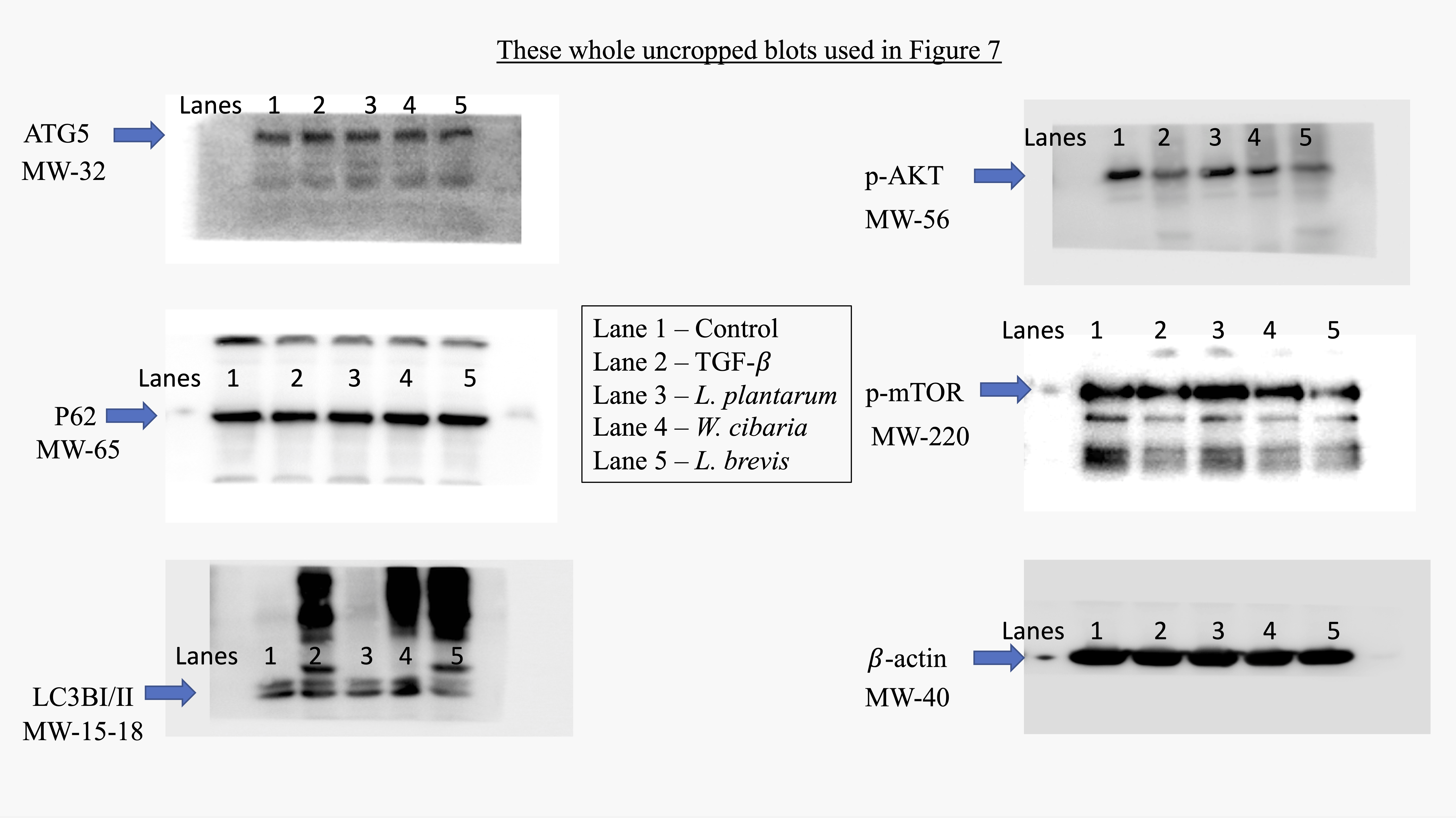

Supplement: S1 Raw images — (ZIP) [file pone.0262767.s003.zip › Uncropped blots_Figure 7.tiff]
