# Supplementary material for: Probiotics counteract the expression of hepatic profibrotic genes via the attenuation of TGF-β/SMAD signaling and autophagy in hepatic stellate cells
Source: PLoS One. 2022 Jan 20;17(1):e0262767. doi: 10.1371/journal.pone.0262767 (PMC8775563; doi:10.1371/journal.pone.0262767)
Supplement: S1 Table — (DOCX) [file pone.0262767.s002.docx]

**S1 Table. List of primers used for RT-PCR.**

**Target Primer sequences Annealing**

**Gene Temperature**

β-actin F5-CAAGAGATGGCCACGGCTGCT-3 63^o^C

R5-TCCTTCTGCATCCTGTCGGCA-3

IL-6 F5-CATCCTCGACGGCATCTCAG-3 63^o^C

R5-GCTCTGTTGCCTGGTCCTC-3

CXCL8 F5-CTGGCCGTGGCTCTCTTG-3 63^o^C

R5-CCTTGGCAAAACTGCACCTT-3

CCL2 F5-CTCAGCCAGATGCAATCAATG-3 63^o^C

R5-AGATCACAG CTTCTTTGGGACAC-3

IL-1β F5-GTG GCA ATG AGG ATG ACT TGT TC-3 63^o^C

R5-TTG CTG TAG TGG TCG GAG-3

SMA-α F5-TGT GAA TGT CCT GTG GAA TTA TGC-3 62^o^C

R5-ACA CAT AGG TAA CGA GTC AGA GC-3

TIMP1 F5-GCC CAG AGA GAC ACC AGA GAA C-3 64^o^C

R5-CTA TCA GCC ACA GCA ACA ACA GG-3

TIMP2 F5-GCA GGA GGA ATC GGT GAG GTC-3 64^o^C

R5-ACA GGC AAG AAG CAA TGG CAA C-3

Col1A1 F5-CGG AGG AGA GTC AGG AAG-3 58^o^C

R5-ACA CAA GGA ACA GAA CAG TC-3

MMP2 F5-TGA CGG TAA GGA CGG ACT C-3 60^o^C

R5-ATA CTT CAC ACG GAC CAC TTG-3

ICAM1 F5-ATG CCC AGA CAT CTG TGT CC-3 60^o^C

R5-GGG GTC TCT ATG CCC AAC AA-3

Lox F5-TCG CTA CAC AGG ACA TCA TGC-3 63^o^C

R5-CAA TGG ATA AAT CAG TGC CTG GTG-3

LoxL2   F5-GGA AAG CGT ACA AGC CAG AG-3 60^o^C

R5-GCA CTG GAT CTC GTT GAG GT-3
