# Supplementary material for: Probiotics counteract the expression of hepatic profibrotic genes via the attenuation of TGF-β/SMAD signaling and autophagy in hepatic stellate cells
Source: PLoS One. 2022 Jan 20;17(1):e0262767. doi: 10.1371/journal.pone.0262767 (PMC8775563; doi:10.1371/journal.pone.0262767)
Supplement: Attachment — Submitted filename: Reviewer response_PLos One 2.docx [file pone.0262767.s005.docx]

**Response to the Reviewer**

Reviewer #1:

In question 7, the authors analyzed mRNA level (48 hours) and protein expression (120 minutes). Regarding the central dogma, transcription will take place first and then translation. Could the authors consider this issue for analysis of mRNA and protein level to determine the time points?

Answer: First, we would like to thank for your valuable review and your suggestions. Several studies used similar time points for the mRNA level and protein level study in the TGF-beta mediated inflammation and fibrogenesis in vitro (Mei et al., 2011 PLoS ONE 6(12): e28915, Zhu et al., 2011, Mole Med rep 4: 505-509; Lynch et al., 2012; Carcinogenesis, 33:976-985; Linag et al., 2013, Biochem Pharmacol, 85: 1594-1602; Song et al., 2021; [Exp Ther Med](https://www.ncbi.nlm.nih.gov/pmc/articles/PMC7967870/), 21(5): 420). We also published several papers with the similar time points.

In question 8, it is fully understandable that the first author is gone, however, it can not be a reason why the information is not showing in the figure. Please provide and repeat total form of those protein markers.

Answer: Thank you for your concern. We express our sincerest apology and are sorry about inconveniences to the reviewer. However, we’d like to show that other several studies in renowned journals also used beta-actin as alternative to compare the phosphorylated form of proteins without total forms. (Zhang et al. J Biol Chem . 2015 Mar 27;290(13):8232-42., Csati et al/ J Headache Pain . 2015;16:99., Sizemore et al. J Biol Chem . 2002 Feb 8;277(6):3863-9.) We hope the reviewer to understand our situation that additional experiment is not be able to be performed and to accept our apologies.

Reviewer #2:

The authors addressed all my questions and made changes accordingly. important materials are added. There's no further questions from me.

Answer: Thank you so much.
